# Prevention and Arrest of Epidemic Disease in Wartime

**Published:** 1917-02-24

**Authors:** 


### Prevention and Arrest of Epidemic Disease in Wartime.

Under this title Lieutenant-Colonel Monckton Copeman, M.D., delivered an interesting lecture at the same meeting. He exhibited on the screen charts showing the number of deaths and incapacitations from missiles as compared with those due to disease, indicating that the medical and sanitary work in the present war was more successful than in the South African campaign, both as to proportionate admissions to hospital, and as to deaths. The incidence of typhoid fever was 33 per 100,000 in 1870-80, 20 in 1880-90, but only 4 per 100,000 in 1915. At the outbreak of war attention was concentrated upon the detection and isolation of any carrier cases in the cook-houses. In his own Division, during the first eighteen months of the war, there was only one case of enteric fever, and that case had refused to be inoculated, and tried to persuade others to refuse also. Compared with the figures for typhoid in the South African War, the proportion of cases of that disease was now $\frac{1}{140}$th.

Speaking of paratyphoid A and B, he said the earliest cases of this disease occurring in France were in Indian troops, or those attached to Indian Divisions. In Gallipoli and Lemnos, paratyphoid was responsible for 93 per cent. of the 5,000 cases of enteric fever. He passed on to speak of the various methods of purifying the water supply, and of the other means of food contamination besides water, such as flies and dust-storms. He showed two remarkable photographs of flies feeding on sugar. The first pictured clearly the proboscis of the fly producing the fluid transparent bladder on the sugar, through which it sucked the sugar; the second showed this bladder black, as the fly had been previously feeding on filth, and it also defæcated on the sugar.

In regard to cerebro-spinal meningitis, he mentioned that thirty-seven district laboratories were set up in the country, and a motor laboratory was fitted up so as to deal efficiently with outbreaks at a distance. He explained the methods of dealing with the cases, and the examination of contacts. The lecturer thought that although typhus fever—the disease of overcrowding, dirt, and destitution, had not been experienced, and was not present in England, we must be on the look-out for it among the troops returning from the war. The tale was quite different in Serbia during 1915, owing to the gross overcrowding of the hospitals and the destitution of the people generally. In its spread the intermediary was the body-louse, a creature which was readily killed out by the appropriate measures.

